# The relation between avian coronaviruses and SARS-CoV-2 coronavirus

**DOI:** 10.3389/fmicb.2022.976462

**Published:** 2022-10-13

**Authors:** Hanan Al-Khalaifah, Mohammad Alotaibi, Afaf Al-Nasser

**Affiliations:** Kuwait Institute for Scientific Research, Kuwait City, Kuwait

**Keywords:** avian, coronavirus, MERS, CoV, SARS-CoV-2

## Abstract

The coronaviruses (CoVs) are a family of ribonucleic acid viruses that are present in both mammals and birds. SARS-CoV and MERS-CoV originated in bats, and there is a possibility that this could be the case for SARS-CoV-2 as well. There is already evidence that a probable intermediary host is responsible for the emergence of viruses in humans as was the case for SARS-CoVs and MERS-CoV. As the SARS-CoV-2 originated from a live animal market, there is always the question if domestic animals are susceptible to these viruses and the possible risk of zoonotic transmission with mammals, including humans. This uncertainty of the transmission of the COVID-19 virus between humans and animals is of great significance worldwide. Hence, this paper focuses on the avian CoVs and their possible relation and interaction with SARS-CoV-2.

## Introduction

Coronaviruses (CoVs) are the largest known RNA viruses with a genomic length of about 30 kb. Structurally they are spherical with a diameter between 50 and 200 nm and spiked bulbous projections of S membrane glycoprotein mediating fusion with target cell membranes (Brown et al., 2016; Uyanga et al., 2021). CoVs belong to the subfamily *Coronavirinae*, family *Coronaviridae*, and order *Nidovirales*. The most distinctive feature of *Coronaviridae* is the genomic size, with the largest genomes among all RNA viruses (26.4–31.7 kb in length) with a G + C (guanine and cytosine) content varying from 32 to 43% (Leila and Sorayya, 2020). In 1937, the Avian infectious bronchitis virus (IBV) was first isolated from an outbreak in chicken flocks. CoVs are a family of enveloped single-stranded RNA viruses of medical and veterinary importance that infect mammals and birds, causing respiratory or enteric diseases. CoVs are members of the subfamily *Coronavirinae* in the family *Coronaviridae* and *Nidovirales* (Li et al., 2020).

Based on their genotype and serology CoVs are divided into four genera: alpha and beta (mostly in mammals), gamma (in birds and marine mammals), and delta found mainly in birds and swine. The 5′ end of the CoV genome encodes proteins required for viral RNA synthesis. A majority of these proteins are encoded by open reading frames (ORFs), ORF1a and ORF1b, which are translated as polyproteins pp1a and pp1ab and are processed by proteinases into 15 or 16 non-structural proteins (NSPs). The 3′ end of the genome encodes structural proteins: spike (S) glycoprotein, membrane (M) glycoprotein, small envelope (E) protein, and phosphorylated nucleocapsid (N) proteins. The S protein, which binds to specific cellular receptors, is divided into the S1 subunit and the S2 subunit. This protein is essential for T cell responses and induced virus-neutralizing antibodies (Han et al., 2020; Uyanga et al., 2021).

Coronaviruses have been found in numerous animals (i.e., all vertebrates except humans) and are responsible for a range of illnesses, which are respiratory, enteric, hepatic, and neurological. These viruses are also the causative agents for common colds in humans. Other members of coronavirus in beta-genus have caused outbreaks of fatal diseases such as the severe acute respiratory syndrome (SARS, caused by SARS-CoV) in 2002–2003, and the recent Middle East respiratory syndrome (MERS, caused by MERS-CoV) in 2012. SARS and MERS are zoonotic pathogens since they originate from animals as a result of transmission to humans (Wille et al., 2016; Suryaman, 2021).

The last 20 years have seen a surge in viral diseases such as SARS-CoV, MERS, and the recent pandemic virus, SARS-CoV-2. The COVID-19 outbreak, which originated from a wet animal market in China in December 2019, and caused alarming infection and death rates, has escalated to a global pandemic. The SARS-CoV-2, which is from the beta group, shares an 89% nucleotide identity with bat SARS-like-CoVZXC21 and 82% with that of human SARS-CoV. It was also found to be 96.2% similar to the coronavirus whole genome in bats, 79% similar to the SARS-CoV genome, and 50% similar to MERS-CoV (Tang et al., 2020). However, the amplifying mammalian host, intermediate between bats and humans, is not known. The first cases of COVID-19 were identified from the wholesale market in Wuhan, China, the epicenter of the virus; animal-to-human transmission was thought to be the mode of transmission, although later cases have shown human-to-human transmission as well (Cascella et al., 2020).

### A general view on avian coronaviruses

In 2018, the International Committee on Taxonomy of Viruses (ICTV) decided to remove all formerly recognized different avian Gammacoronaviruses and allow only “avian coronavirus (ACoV)” as the official name of the species in the genus Gammacoronavirus. This meant that all Gammacoronaviruses irrespective of the bird species would be considered to be the same species of the virus even if they have different hosts and tropisms, different antigenicity, lack of cross-protection, and differences in genomes (de Wit and Cook, 2020).

A large number of CoVs have been detected in avian species. Attention to the occurrence of CoVs in other species like birds has gained a lot of attention since the breakout of SARS-CoV in 2002. Previously, knowledge of CoVs in avian species was restricted to three birds of the order Galliformes, i.e., domestic fowl (Gallus gallus), turkeys (genus Meleagris), and pheasants (Phasianidae), with their IBV, turkey coronavirus (TCoV), and pheasant coronavirus (PhCoV), respectively. For a long time, these three viruses were regarded as different species due to their diverse pathotype, host range, and genetic relatedness of the S protein. All viruses found in birds like penguins, pigeons, peafowl, parrots, waterfowl, teal, quail, duck, and whooper swan are classified as avian CoVs within the subgenus Igacovirus of the genus Gammacoronavirus (Decaro and Lorusso, 2020).

Wild birds are natural hosts for many zoonotic pathogens and can directly or indirectly affect human health. They also represent one of the microbial pathogen transmissions to domestic animals and infections that lead to socio-economic repercussions. Wild birds are mostly associated with the transmission of influenza A viruses and are also thought to be responsible for the spread of the West Nile virus *Borrelia burgdorferi*, and also bacterial infections such as *Salmonella* and *Campylobacter*. Decade-long studies have proven the existence of CoVs in wild birds. The other factors that also make birds a good source for various pathogens are their rich biodiversity pool of species, ecological traits like gathering during feeding and roosting and also flying long distances passing through different environmental climates. Avian CoVs are the primary members of the genus Gammacoronavirus including IBV, which affects chickens, and other viruses isolated from domestic Galliformes such as TCoV, responsible for turkey enteritis, and the guinea fowl coronavirus (GfCoV). Viruses were also discovered in pheasants, peafowl, quails, and non-Galliformes, namely Columbiformes, Pelecaniformes, Ciconiiformes, Psittaciformes, and Anseriformes. CoVs detected in birds of the Passeriformes order, such as munia, bulbul, and thrush, were found to be unique to the already existing CoVs, and this was the deciding factor for the generation of the genus Deltacoronavirus (Miłek and Blicharz-Domańska, 2018).

### Animal models for SARS-CoV and SARS-CoV2

Substantial trials have been conducted in different domestic and laboratory animals and birds to assimilate the clinical manifestation of SARS-CoV and SARS-CoV2. However, the majority of animal models showed vast variations in their clinical manifestations. It is essential to envisage the major criteria that denote the animal models as suitable candidates for a given human disease before reviewing the currently available data about the experimental trials.

Animals become an appropriate model if they can represent certain aspects of human disease complexity. The animal model candidate does not necessarily mimic the entire human disease complexities rather than specific facets of the disease. There are five criteria for selecting an animal model (Denayer et al., 2014). These are: (1) Species: the species that are capable of depicting the pathophysiology of the disease close to that reflected in humans are more suitable candidates; (2) Complexity: the model needs to reveal the complexity of disease in the utmost detail; (3) Disease simulation: the simulation of the disease could be subjected to several pathways and principles. Hence, the model should exploit the complexity of the disease through collective pathways and consequences; (4) Predictivity: a criterion that is mainly applied for assessing the effect of the drug on the outcome of the disease; and (5) Face validity: this criterion defines the extent of the model in reflecting a symptom or set of symptoms.

Animal models are classified into groups according to their biological, genetic, and pathophysiological requirements. They are as follows: (1) Induced experimental models: certain impairments experimentally induced to allow their study; (2) Spontaneous genetic/mutant models: naturally existing genetic variants that allow the study of the side effects of mutation; (3) Genetically modified models: modification induced by genetic engineering and embryo manipulation; (4) Negative models: species or breeds that are naturally resistant to certain infectious diseases; and (5) Orphan models: non-human species that suffer from certain natural functional disorders. The susceptibility of several domestic and laboratory animals was tested for the expression of the patho immunogenicity of SARS-CoV and SARS-CoV2 (Denayer et al., 2014).

### The avian coronaviruses–Infectious bronchitis virus

It is well known that the Coronavirinae subfamily possesses a very large RNA genome. The ACoV is about 27,500 nucleotides (nt), smallest is 27,231 nt of the IBV strain. Duck CoV has a similar size of 27,754 nt (Miłek and Blicharz-Domańska, 2018). The ACoVs have a positive-sense and single-stranded RNA genome of approximately 27.6 kb in length and encode the N, M, E, and S proteins (A Rohaim et al., 2019). Avian IBV is a gamma-coronavirus in the family *Coronaviridae*, the order *Nidovirales*, and the genus *Coronavirus* that causes a highly contagious upper-respiratory disease in domestic chickens. In layer-type birds it can cause a drop in egg production and some strains are nephropathogenic. Infectious bronchitis remains one of the most widely reported respiratory diseases of chickens worldwide despite the routine usage of attenuated live vaccines to control the disease. Control of IBV is difficult because there is little to no cross-protection between the numerous different serotypes of the virus (Thor et al., 2011).

Infections occurring due to the ACoVs are responsible for acute, highly contagious, and economically important diseases in poultry. It was first identified in ND, USA, and then spread rapidly around the world. Indirect transmission of these viruses is suggested from contaminated litter, footwear, clothing, utensils, equipment, and personnel (A Rohaim et al., 2019).

In chickens (*Gallus gallus*), the IBV is the only Gammacoronavirus that is well-studied and is the known cause of diseases that affects socioeconomic welfare (de Wit and Cook, 2020). IBV infection on a global scale has been estimated to be the second most dangerous poultry disease after highly pathogenic influenzas. The ill effects caused by the IBV strain can be due to climate, dust, ammonia, density, and cold stress. Other factors such as the age of the bird, its immune status, and the presence of secondary or co-infections also play a role. The mortality rate caused solely due to IBV infection is low but becomes a cause for post-secondary infections with bacteria such as *E. coli*. There have been no known effects on human health. However, there is an urgent need to control IBV due to the negative influence of the use of antibiotics and the development of antibiotic resistance. There is a hike in demand for antibiotic chickens due to which treatment and welfare of diseased birds may be ignored (de Wit and Cook, 2019).

The IBV initially infects upper airway epithelium tissues and based on the type of BV strain, the severity of the disease ranges from mild respiratory disease to kidney failure and death (Parsons et al., 2019). The disease affects the productive performance of both meat and egg-laying birds of all ages. The IBV genome length is approximately 27.6 kb linear, non-segmented, positive sense, single-stranded RNA. The viral genome is 5′ capped, with a poly-A tail, and the gene organization is 5′ UTR-1a/1ab-S-3a-3b-E-M5a-5b-N-3′ UTR. The 5′ two-thirds of the IBV genome encodes two polyproteins 1a and 1ab that contain NSP that are associated with viral RNA replication and transcription, whereas the 3′ one-third encodes four structural proteins: the surface spike glycoprotein (S), small envelope (E), membrane (M), and nucleocapsid (N) proteins (Sun and Liu, 2016).

### Symptoms of coronavirus infection in birds

Overall, it is revealed that the avian CoVs are related to SARS-CoV-2. Many research studies have shown similarities between the two (Iglesias-Osores and Saavedra-Camacho, 2020). However, further studies are required to know how avian CoVs can affect humans and what will be its consequences. Development and production of vaccines can counter pandemics or outbreaks, but continuous mutation of the virus creates a hurdle for the vaccine developers. One good example is IBV, which is found to have a significant mutation rate as compared to other viruses. Another problem is that the poultry industry is facing huge threats to survival. So, scientists must cooperate and perform genetic sequencing and analyses of the IBV with its given structural and non-structural genes. A study conducted in China found that there was a change in four dominant genotypes. The results of the study also revealed that the Southern Chinese region had observed significant changes in the genetic diversity of the virus, which indicated that the virus mutated to come up with new strains. As a result, the previous vaccines were not effective against the mutated virus (Fan et al., 2019).

Chu et al. (2011) noted a high prevalence (12.5%) of novel ACoV in aquatic wild birds. Post-phylogenetic analyses showed that there is a diversity of gammacoronaviruses and deltacoronaviruses among birds. Gammacoronaviruses were found predominantly in *Anseriformes* birds, whereas deltacoronaviruses were found in *Ciconiiformes*, *Pelecaniformes*, and *Anseriformes* birds. There were recurring interspecies transmissions of gammacoronaviruses between duck species. Contrastingly, deltacoronaviruses may have more stringent host specificities.

It was found that different globall strains of IBV have some differences in different countries or cotenants. For example, Taiwan TW-1 and Japan Q1-like IBV strains were found to be more pathogenic and lethal, respectively (Lin et al., 2016). On the other hand, Korean group I is more closely related to the Mass type, while Korean group II is a different branch (Lim et al., 2011), in which the A KM91-like backbone recombinant, and the SNU8067 inhibit the formation of the ovarian follicles and the maturation of the oviduct (Hong et al., 2012). The African strains of IBV were reported to be more virulent in day 1 SPF chickens with 50% fatality (Toffan et al., 2011a). The Chinese isolate QX1 that causes respiratory symptoms were reported in Italy in 2011 (Toffan et al., 2011b). In USA, the IBV Cal99 variant was found to cause inflammation of the neuropathogenic and could reach the gastrointestinal and respiratory tracts, and the bursa of Fabricious (França et al., 2011). In Brazil, it was reported that the presence of IBV was higher in the digestive system at 43.5% and lower in the respiratory system at 37.7% (França et al., 2011).

### Protective measures

Protective measures are taken to minimize and prevent the virus spread among humans. The important step is applying protective measures such as wearing masks, which would highly minimize the viral spread, and governments could mitigate restrictions afterward. As studies have shown, coronavirus in humans is usually spread following a respiratory infection, which then spread after symptoms develop such as sneezing and coughing in closed places. As the vaccine is one of the best methods to prevent diseases and minimize symptoms caused by pathogenic microorganisms like bacteria and viruses, scientists are continuously monitoring viral mutations so that vaccines can be developed with more efficiency. In the previous decades, vaccines have helped the world to control different types of diseases to a great extent (Schervish, 2012).

For the poultry flocks, it is critical to monitor the immune and health status of the birds on regular basis to avoid outbreaks and diseases, including respiratory diseases. Boosting the immune status against infectious agents by using effective feed ingredients such as probiotics, herbal inclusions, and other natural resources is a popular approach to optimize the health and immune status of the flock (Al-Khalaifah and Al-Nasser, 2021; Amer et al., 2021, 2022; El-Maaty et al., 2021; Ibrahim et al., 2021; Al-Khalaifah and Uddin, 2022; Al-Khalaifah et al., 2022a,b; Al-Surrayai and Al-Khalaifah, 2022; Attia et al., 2022; Kishawy et al., 2022; Omar et al., 2022).

### Diagnosis

Vaccination is the most commonly used mean of controlling IBV. Serological diagnoses using ELISA and other commercial kits are also available, but the downside of ELISA is that it is unable to distinguish between the types of IBV. There are methods available to differentiate and classify isolates. The most preferred typing method is molecular genetics by amplifying viral RNA and sequencing based on S1 protein sequence, which is rapid and reliable. Although the detection of viral RNA does not discriminate between infectious and non-infectious viral particles, it detects the desired genome with high specificity and sensitivity. To keep any disease in control, prevention is the best path to take, but there should be a high level of biosecurity, single-age housing, effective cleaning, and sanitization of poultry farms and machinery that are in contact with poultry or poultry litter and the removal of excreta from the surroundings. Inactivated IBV vaccines are not effective if they are not primed with live-attenuated vaccines. In young birds, suitable combinations of live vaccines provide protection against many strains. In older birds, a boost with an inactivated vaccine enhances protection during the laying period. The methods used to administer these live-attenuated vaccines are through a coarse spray, aerosol, or *via* drinking water. At the same time, the method of applying the vaccine such as the quantity, quality, and temperature of water used to dilute the vaccine, dosage, and combination with other vaccines simultaneously or in short intervals can play a negative role in the effectiveness of the vaccine. If the vaccination dose was low and did not produce the anticipated effect, it will cause reduced or delayed protection from the virus (de Wit and Cook, 2019).

Infectious bronchitis virus vaccines were given over the years to the poultry to save the animals, but the high viral mutation rate caused continuous challenges for animal owners and scientists. The continuous mutation allows the virus to escape from the immune system that the poultry acquired following vaccination. Poultry farm owners had to take special measures to protect their animals from viral infection and spread. Following animals’ viral infection, farm owners were required to separate infected animals, to protect and save other animals (Zhang et al., 2021). When symptoms of the ACoV infection show up, farmers need to recognize that their animals have been affected and run strict management. One of the main symptoms in birds and chickens is sneezing and rales. They will also show signs of renal infection. In addition, in hens, poor egg production can be seen. Alloplasia and inflammation have been also been reported in animals infected with IBV. Once these symptoms are visible in the animals, the farmers are responsible for separating these animals from others to minimize and control the spread of the disease on the whole farm (Zhang et al., 2021).

The IBV caused by avian coronavirus undergoes recombination with other strains that lead to mutations. Further studies are required to determine the potential effect of avian CoVs strains on humans, as well as birds, and its consequences in the future for either animals or humans and the economy. In addition, studies are needed to find out to what extent these viruses can be mutated and how the viral spread can be controlled, and its nature, for the development of new efficient viral vaccines (Cavanagh, 2007).

High losses in poultry production inflicted by IBV infection are mainly due to the combination of high morbidity and loss of growth performance, accompanied by secondary bacterial infections (Decaro and Lorusso, 2020). IBV inactivated and live attenuated vaccines are widely produced. The inactivated vaccines are mainly used as a booster in older, egg-laying chickens. Despite the wide application of the IBV-inactivated and live attenuated vaccines, the disease continues to be a major problem for the poultry industry due to the existence of many serotypes. The variations in the surface spike protein denote wide diversity, which leads to poor cross-protection and loss of immunogenicity.

Live attenuated IBV vaccines are produced by repeated passage in embryonated eggs, resulting in spontaneous mutations. As a consequence, attenuated viruses have a small number of mutations, which could be associated with the loss of virulence and/or immunogenicity, with a major risk of reverting to virulence. Wide use of the vaccines contributed tremendously to the high variability of IBV through recombination between vaccine strains and the field viruses, as well as selection pressure due to extensive use of the vaccines, which induces partial immunity in the vaccinated birds. Hence, the continuous generation of new IBV variants due to mutation and recombination leads to great difficulty in controlling IBV outbreaks (Lin and Chen, 2017; Tizard, 2020).

Some other avian species from which CoV has been definitively associated with the disease are turkey, pheasant, and guinea fowl. Turkey’s coronavirus (TCoV) is one of the best described and economically the most important ACoV after IBV. It was identified as early as the 1940s as a cause of enteric disease in the USA and even now affects turkeys of all ages, and the young ones have a high mortality rate in many parts of the world. Some of the indicators of distressed turkeys are reduced feed and water intake, wet droppings, diarrhea, and loss of body weight. It may also cause poultry enteritis and mortality syndrome (PEMS), which is associated with high mortality, growth retardation, and immune dysfunction. In turkeys that are breeding, there is an abnormal egg-laying pattern because of TCoV infection. The replication occurs mainly in the intestinal tract and bursa of Fabricious. Its transmission takes place by fecal or oral route. The association between IBV and TCoV is debatable, as IBV vaccines have sometimes been isolated from turkeys. These two viruses have a similarity index of 80% in some gens and about 35% of similarity in the spike proteins. Although the two viruses originate from different species, genomic studies indicate that the virus might have a common origin (de Wit and Cook, 2020).

The pheasant coronavirus or PhCoV is known to cause respiratory and renal problems and is closely related to TCoV and IBV. PhCoV can replicate in chicken but whether it causes the disease is still unknown. The GfCoV is known to cause acute enteritis with a high mortality rate and pancreatic degeneration. Besides showing similarities to IBV and TCoV, there are differences in the spike gene. In some non-gallinaceous species such as teal and racing pigeons, which were kept near domestic chickens in China and Australia, respectively, IBV strains were isolated. Using RT-PCR, Gammacoronaviruses have been identified in some geese, pigeons, and mallard ducks (Al-Khalaifah et al., 2020; de Wit and Cook, 2020).

### Is it possible for COVID-19 to spread to poultry?

By now, it is known that SARS-CoV2 is a β CoV and IBV is a δ CoV. Although studies have shown that there is recombination between δ CoVs of different avian species and β CoVs in different mammals, proof of recombination between CoVs of different genera is unavailable. Recombination of an α or β CoV is highly unlikely. For recombination to take place, two viruses have to be present in the same cell of the same bird at the same time, which is possible as replication takes place in the same host (de Wit and Cook, 2020). As seen through comparative genome analyses, birds can be a gene source of Gammacoronavirus and Deltacoronavirus, resulting in constant evolution and dissemination of CoVs. However, this does not prove that co-infection facilitates recombination. In a recent study in Brazil, it was reported that a patient had a co-infection of two SARS-CoV-2 variants (Pedro et al., 2021).

As poultry are widely used by humans all over the world and also due to their contact with other mammals in live animal markets, a susceptibility study was conducted with SARS-CoV-2 and MERS-CoV in five common poultry species: chickens (*Gallus gallus domesticus*), turkeys (*Meleagris gallopavo*), Pekin ducks (*Anas platyrhynchos domesticus*), Japanese quail (*Coturnix japonica*), and White Chinese geese (*Anser cygnoides*). For the experiment, 10 birds from each species were challenged with either the USA-WA1/2020 isolate of SARS-CoV-2 (BEI NR-58221) or the FL/USA53 2_Saudi Arabia_2014 isolate of MERS-CoV (BEI NR-50415). Oropharyngeal and cloacal swabs were collected from all birds at 2, 4, and 7 days post-challenge (DPC) and were tested for virus by RT-PCR. On the 14th day, sera from birds were collected and tested for antibodies. There were no clinical signs or virus detected in swabs; antibodies also were not detected in serum, which showed that the virus did not replicate in any of the poultry species (Suarez et al., 2020).

From an economic perspective, the COVID-19 pandemic has forced the closure of non-essential businesses to lower the transmission rate of the disease. However, food production units including dairy and poultry are still required to function to meet the food demands of consumers, but not without repercussions. A Canadian study reported that there was an increase in the sale of fresh chicken by 50% in mid-March of 2020 in comparison with the sales record of the previous year for the same week. The consumption pattern of eggs also changed, before the outbreak, 70% of eggs consumption was in shelled eggs and the rest was through breaker eggs, eggs in liquid form. Also, due to the shutdown of most hotels, restaurants, and convention centers, demand for breaker eggs dropped between 40 and 60% in the 3 weeks up to April 7th. In the United States, the prices for chicken parts also dropped but wholesale prices for eggs tripled from mid-March to early April.

Probably one of the greater impacts of the outbreak has been on the availability and cost of certain feed ingredients such as dried distillers grain (DDGs) for animals. An increase in DDGs results in changes in the least-cost ratio and feed costs for animal farmers (Weersink et al., 2020). The dairy and poultry farms continue to operate but with strict health measures and social distancing. The consequences of not adhering to the safety protocols against the virus were seen in the case of Luciana Viviana da Silva, a 58-year-old female worker who died from the virus in a poultry factory in Northern Ireland. The workers union Unite has urged the local government to take strict measures after the incident, which resulted in the shutting down of the factory. They have asked for all employees and their families to be tested and for workers to receive full pay for the time they worked. Following this incident, other meat processing factories have taken measures such as the installation of Perspex screens, a social distancing between workers, thorough cleaning and use of personal protective equipment (PPE), and incentives for all employees (McCullough, 2020).

Poland is Europe’s larger broiler meat producer. Statistical analysis has shown that the EU price for broiler meat has considerably dropped by 8.4% and in Poland by 37%. Based on the reports from the Polish National Poultry Council–Chamber of Commerce (KRD–IG), the outbreak has resulted in 40 million broilers in the domestic market due to export restrictions, which has caused an oversupply. With so many surpluses, it has become extremely difficult to sell eggs, chicks, or raised birds outside Poland, which could further result in huge economic losses. Not only exports but also domestic markets have been severely affected (Vorotnikov, 2020). Certainly, there have been clear and significant losses to dairy and poultry supply chains due to COVID-19, but the structure of supply management in dairy, poultry, and eggs may help industries come out faster from these hurdles (Weersink et al., 2020). SARS-CoV-2 was transmitted at the beginning from bats to humans, then between humans, and later was found in animals worldwide, which was suspected to be transmitted from humans to animals. These animals included gorillas, pumas, pet dogs, cats, snow leopards, tigers, lions, and mink (Iglesias-Osores and Saavedra-Camacho, 2020; Sharun et al., 2021; Wacharapluesadee et al., 2021).

Pandemics are frequently caused by a group of viruses called coronavirus that cause infections in birds and mammals (Sahin et al., 2020). The high capacity of these viruses to infect humans, such as in the case of SARS or MERS, has become highly relevant in the outbreak of a new coronavirus disease (COVID-19) in a market in the city of Wuhan, China, and its spread throughout the Asian continent and later to more than 180 countries in the world, causing a pandemic. The CoVs also cause diseases in mammals such as dogs, mice, horses, whales, and cats, in animals of economic importance and global consumption such as birds (including poultry such as broilers and turkeys) (Biswas et al., 2020). Birds are possible reservoirs of SARS-CoV-2 and can transmit it to humans or vice versa. COVID-19 is caused in humans by Betacoronavirus SARS-CoV-2; in poultry, CoVs cause Avian Infectious Bronchitis by Gammacoronavirus, which produces a highly contagious disease in chickens (Gorbalenya et al., 2020).

Coronaviruses, in general, are spread throughout the planet and are highly infectious, in addition to being extremely difficult to control because they have high genetic diversity over large areas, short multiplication periods, and a high rate of mutation (Sahin et al., 2020). SARS-CoV-2 uses a host cell receptor angiotensin-converting enzyme II (ACE2), and IBV enters the host cell similarly to other CoVs by the attachment of the S1 protein as a receptor binding subunit that consists of N-Terminal domain (S1-NTD) and C-Terminal Domain (S1-CTD) as a receptor binding domains (RBDs). These domains start attachment to the host cell receptor and the S2 protein is responsible for viral-cell and cell-cell fusion. The IBV receptor in the avian cells was reported to be α-2,3 linked sialic acids unit that is attached by the S1 subunit. Both of these S1 subdomains, NTD and STD that are RBD binds to different sugars and proteins host receptor (Winter et al., 2006). In addition, host heat shock protein member 8 (HSPA8) was reported to be a factor of IBV infection (Zhu et al., 2020). It is reported that furin is responsible for S protein cleavage to S1 and S2 that promote IBV viral entry to host cells (Yamada and Liu, 2009; Liu et al., 2021). It is also clathrin-mediated endocytosis and requires a classical endosomal/lysosomal system. IBV produces respiratory tract infection, and affects the reproductive tract, and some strains can cause nephritis; SARS-CoV- 2 cause SARS. IBV genotypes and serotypes are related to the vaccine strains (S1) and SARS-CoV-2 does not have a variant yet. The IBV incubation period is very short compared to SARS-CoV-2, which is 18–36 h and depends on the dose of virus infection, and clinical signs appear within 24 and 48 h of exposure to the virus. Information about zoonotic reservoirs and their transmission among them can help understand the COVID-19 outbreaks and zoonotic transmission of IBV. There should be a clear knowledge of the reservoir host, distribution pattern, and spreading routes of IBV. SARS-CoV-2 does not have a probability of infecting chickens or any other poultry; the main reason for non-infection in birds is that both viruses have different receptors on the hosts and belong to phylogenetically different groups.

### Coronaviruses on surfaces

The most critical situation for either animal owners or governments is to identify different types of surfaces that help viruses to survive for a certain period and spread. Hence, upon contact with these surfaces or fomites contaminated with the pathogenic virus(s), they have the probability of infecting new hosts. Research studies have reported the existence of several routes of viral transmission between animals or between humans. The common surfaces found to be polluted are doorknobs, light switches, and remote controls, which allow the viral spread among humans, while glass and stainless steel can cause the viral infection and spread either between animals or humans (Tiwari et al., 2006; Vasickova et al., 2010). The other critical part is that the virus can be infective for days, having the capacity to potentially infect humans or animals for a longer time. Essential management includes disinfecting these surfaces continuously to stop and eliminate any chance of viral infection and spread. Surfaces contaminated with the virus are required to undergo virological monitoring continuously, to determine the reasons behind the virus spread. This is how environmental and physical risks of pathogenic viruses on different surfaces can be identified to make better and more proactive decisions to deal with a virus (Vasickova et al., 2010).

Researchers have also performed and described several methods for humans to inactivate viruses, by disinfecting surfaces and remaining proactive to minimize the threat of a virus in a given system. By cleaning the surfaces using different types of disinfectants, the chances of viral spread can be minimized. When people are infected with a virus, it is important to be isolated to prevent viral spread. Studies of viral mutation are important to find more data on the extent to which viruses mutate and the spread of a disease that can be controlled, and the nature of the virus (Konkolova et al., 2020). A research study by Zou et al. (2013) team reported that the avian influenza virus can be inactivated by using certain disinfection measures, using chemical treatments under different physical conditions. Their study was performed on two viruses, using different disinfection methods such as changing pH, ultraviolet light, and different temperatures. The results showed that both viruses were able to sustain until a certain level of temperature and ultraviolet light. When the recommended concentration of ethanol, Virkon^®^-S, and sodium hypochlorite was used, viruses were completely inactivated from the treated surfaces (Zou et al., 2013).

Interestingly, a study by Rulli et al. (2021) team was performed to determine the high-risk hotspots locations of zoonotic diseases such as SARS-like CoV in humans. They investigated the horseshoe bat locations for hotspots to locate beginning of infection from animals to humans. The authors used a number of factors such as; population and livestock density, forest, cropland and human residents cover. These factors were implemented it in an algorithm calculation to determine which locations at high-risk (Rulli et al., 2021). They found that China is a global hot spot and indicated that south shanghai at high risk of becoming a hot spot of zoonotic viral disease spread. In addition, the authors reported that other regions in other countries such as Java, Bhutan, east Nepal, northern Bangladesh, the state of Kerala (India) and Northeast India are hotspots (Rulli et al., 2021). They also reported that Northeast India and Bangladesh regions were hotspots which were known for the previously outbreaks of Nipah virus.

SARS-CoV-like CoV was detected in dead Malayan pangolin lungs (Liu et al., 2019). Another study of Pangolin-CoV reported that over 91 and 90% identical to SARS-CoV-2 and BatCoV RaTG13, respectively (Zhang et al., 2020). They concluded that pangolin is a natural reservoir of SARS-CoV-2-like CoVs. Also, since SARS-CoV-2 identical by 96% whole-genome identity with a bat CoV BatCoV RaTG13 (Zhang et al., 2020). SARS-CoV-like virus transmission between animals that is highly thought to be hybridized to be SARS = CoV-2 and infected humans in late 2019.

China is known to be a large domestic and worldwide producer of poultry like chicken and ducks. Duck which is known to be a reservoir of influenza A viruses including H5N1. Both of domestic and wild ducks barely show symptoms or don’t show symptoms of infection at all. vaccination of ducks against AIV was reported to be non-protective against the virus (Li et al., 2019). That said, spread of AIV and mutation are a highly potential hazard of continuous viral spread.

U.S. Department of Agriculture (USDA) reported over 40 million birds in 36 states were infected with highly pathogenic avian influenza (HPAI) on 2022 (Chanda and Joshi, 2022). Both of the wild and backyard flocks infected with HPAI virus. Although two humans were infected 1 in USA and 1 in UK, it represents a potential hazard of spread since a high number of flocks are infected and represent a danger of viral spread at any moment (Chanda and Joshi, 2022). Thus, continuous monitoring of many potential hazardous viruses, e.g., SARS CoV. AIV, IBV strains and others is highly important to safe guard humans and animals from zoonotic diseases that could start global pandemic similar or worse than SARS-CoV-2 virus and wipe many lives, unfortunately.

## Conclusion

Discovering possible sources or hosts for the SARS-CoV-2 virus is crucial for preventing further infections and for maintaining and ensuring a safe and stable food supply. Both avian and mammalian CoVs are genetically and antigenically different. It is highly unlikely that poultry will act as a source for transmission of these CoVs. To date, there is no evidence to show that there is a possibility of cross-species transmission, although the SARS-CoV-2 transmitted from bats to humans!

## Author contributions

MA contributed to the draft reviewing and editing. All authors contributed to the article and approved the submitted version.
